# The Role of Artificial Intelligence in Fighting the COVID-19 Pandemic

**DOI:** 10.1007/s10796-021-10131-x

**Published:** 2021-04-26

**Authors:** Francesco Piccialli, Vincenzo Schiano di Cola, Fabio Giampaolo, Salvatore Cuomo

**Affiliations:** 1 Department of Mathematics and Applications “R. Caccioppoli”, University of Naples Federico II, Naples, 80126 Italy; 2 Department of Electrical Engineering and Information Technology, University of Naples Federico II, Naples, 80125 Italy

**Keywords:** Artificial intelligence, COVID-19, SARS-CoV-2, Healthcare, Machine learning, Deep learning, Review, Survey

## Abstract

The first few months of 2020 have profoundly changed the way we live our lives and carry out our daily activities. Although the widespread use of futuristic robotaxis and self-driving commercial vehicles has not yet become a reality, the COVID-19 pandemic has dramatically accelerated the adoption of Artificial Intelligence (AI) in different fields. We have witnessed the equivalent of two years of digital transformation compressed into just a few months. Whether it is in tracing epidemiological peaks or in transacting contactless payments, the impact of these developments has been almost immediate, and a window has opened up on what is to come. Here we analyze and discuss how AI can support us in facing the ongoing pandemic. Despite the numerous and undeniable contributions of AI, clinical trials and human skills are still required. Even if different strategies have been developed in different states worldwide, the fight against the pandemic seems to have found everywhere a valuable ally in AI, a global and open-source tool capable of providing assistance in this health emergency. A careful AI application would enable us to operate within this complex scenario involving healthcare, society and research.

## Introduction

COVID-19 may be considered the first influenza pandemic to be disseminated in our hyper-connected world. It has proven to be a phenomenon that significantly and rapidly impacts many layers of our society. Despite the many containment measures adopted to limit COVID transmissions, such as the closing of borders and the introduction of periods of lockdown, we are witnessing as many as 116 million confirmed cases and more than 2 million deaths in 235 different countries, as reported by the World Organization Health (WHO) at the end of February 2021. Serious concerns about healthcare systems’ capacity have arisen due to the unprecedented demand for health services, especially concerning disadvantaged states. In this scenario, methodologies able to speed up diagnostic procedures, enhance monitoring and tracking capabilities, predict the evolutionary stages of the contagion as well as its effects on society, and simulate the results of a containment strategy, a medical protocol or a new molecule, can represent a revolutionary milestone in the progress of the world in facing these dramatic events.

The COVID-19 emergency has given an incredible boost to the improvement of existing models and the development of new prototypes in order to achieve promising results in fields such as the tracing of the infection (Pinotti et al. 2020) or the prediction of its diffusion and the effects of restrictive measures (Della Rossa et al. 2020).

**Fig. 1:**
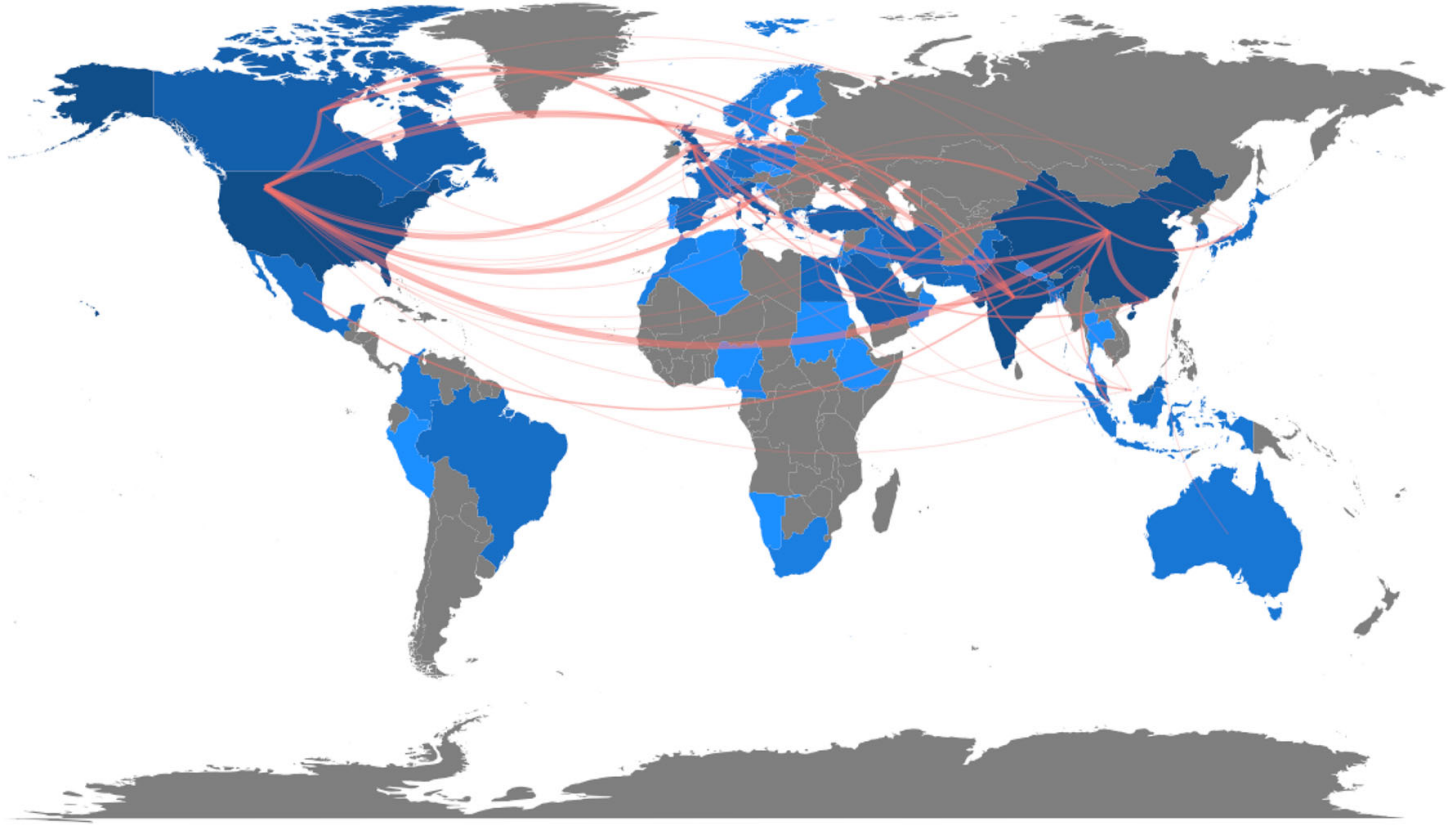
A Worldwide map of scientific production on topics related to AI and the COVID-19 pandemic. Countries with a higher number of peer-reviewed publications are reported in blue; the darker the colour, the greater the number of papers. The links in red represent international collaborations

Advances in AI are expected to represent an effective strategy to face these challenges: thanks to the massive amount of information made available through the advent of pervasive IT, and the continually increasing computational power, AI has shown an outstanding performance concerning most of the problems mentioned above. Indeed, its ability to extract patterns and relations from data has made this research area particularly attractive in tasks involving the description of complex information and dynamics. Successful applications of Deep Learning (DL) (Shorten et al. 2021) and Machine Learning (ML) (Nayak et al. 2021) techniques in image recognition and segmentation, time series forecasting, sentiment analysis, system control and dynamics simulation are widely present in the literature, as well as robotic self-operating solutions that have proven to be effective in containing social contacts. All these promising outcomes explain the great attention focused on worldwide research on AI as an instrument to fight the COVID-19 pandemic. According to the Scopus^1^ database, more than 1000 peer-reviewed papers containing the keywords “COVID-19” and “Artificial Intelligence” have been published from the outbreak of the epidemic to 1st March, 2021. As reported in Fig. 1, even though, not surprisingly, most of this scientific production come from “first-world” nations, these topics have also aroused considerable interest in countries from mid-east Asia and North Africa. For instance, in states such as Iran, the COVID-19 pandemic has struck with particular strength due to a deficiency in monitoring actions and a weaker healthcare system. AI technologies, expecting to offer efficient and low-cost models to address medical and social issues, have naturally attracted many of the more seriously affected regions. Countries such as Iran, Turkey and Egypt, alongside the USA, China, India, Italy and the UK, are among the top ten states where more peer-reviewed papers related to the application of AI technologies to COVID-19 issues have been written.

Moreover, such an observation can provide a perspective on the global dimension of pandemic research. The extensive expertise in AI methodologies of the USA on the one hand, and the great availability of data in China, the country which witnessed the first incidence of the epidemic, on the other, may explain the central role of these countries in the worldwide scientific production. In our opinion, the scenario is complete: the direct impact of the pandemic drives scientific research towards AI methodologies applied in COVID-19 related situations, and collaborations between different countries are strengthened by the common interest in finding effective strategies to contain the crisis in many different fields. A notable literature review of AI tools during the COVID-19 pandemic can be found in Chen et al. (2020).

Such a wave of interest confirms the importance of new strategies in accelerating the development of knowledge and solutions to the present medical and socio-economic emergency. However, some questions related to the role of AI during a pandemic remain open: for example, “Has AI proven itself ready for such a situation?”; “What is the real importance of data, i.e. the importance of their collection and quality in the application of AI techniques to real problems?”; and “Has AI been successfully applied in real COVID-19 situations and what benefit has it brought in these contexts?”. We stated just a few of the questions that this work investigates and discusses by analyzing an extensive collection of recent studies. This article proposes a perspective from a data and applications point of view, framing the problem as a timeline, obtained by reorganizing the pandemic’s temporal phases, as provided by the WHO.

## The Pandemic Dynamics: a Conceptual Overview

The pandemic dynamics landscape is composed of several complex interconnected relations among data, models, and applications, just like a tangled skein of wool. We aim to untangle such dynamics by freezing the pandemic in a temporal stack. This re-organization will simplify the discussion and will reveal pandemic-related AI research trends, as well as the evolution of new data and DL models.

AI is usually applied as a data-driven approach to complex problems since the relations involved are usually hard to describe by mathematical or statistical models. It means that the type of data strongly influences the AI methodologies to be adopted in a specific context. Table 1 offers the reader a summary of these different kinds of data and the tasks that are addressed in each context, and that can be found in the scientific literature.

**Table 1:** Schematic overview of information types, related sources, applications and AI methods

Data type	Multimedia content	String patterns	Time series
Description	Image, Video, Audio, 3D Data Points	Texts, Genetic Sequences, Semantic Networks	Historical Data, Sensor Data, Scientific Data
*Sources*	Medical Images (CT, X-ray), Lung Ultrasound (LUS), Surveillance and Security Cameras, Drones, Acoustic Data, Smart Speakers, 3D Protein	Log Data, Documents, Scientific Literature, Genome Maps, Social Network Messages, Electronic Health Records	Clinical Values, Wearable Device, Internet of Things (IoT) Data (ventilators parameters, etc.), Statistical Parameters (deaths, ICU occupancy), Economic Indicators (GDP)
*Examples*	Computer Vision, Motion Detection, Voice Recognition	NLP, Classifying Reviews	Sensor Data, Web Activity
*Application*	Medical Disease Detection and Diagnosis, Social Monitoring, Proteomics	Social Monitoring, Medical Treatments, Pathogenesis	Healthcare Optimization, Social Planning, Epidemic and Transmission Prediction
*Examples*	Pneumonia Diagnosis, Mask Detection, Environment Recognition, Social Control, People Displacement Analysis, Disinfection Robots, Quarantine Checking, Cough Sound Recognition, Protein Folding	Medical Protocols Enhancing, Drug Development and Repurposing, Vaccine Development, Genomics	Outbreak and Transmission Prediction, Infection Tracking and Prediction
*ML/DL Tecnique*	DeCoVNet, FCNN, ResNet-18/34/50, SqueezeNet, DenseNet-161, VGG19, STN, CNN, SVM,RF, MLP, Grad-CAM U-Net, FCN SegNet, DeepLabv3	ML-DSP, NLP, RNN, RF, LSTM, SDV, LDA	PNN+cf, BFGS-PNN, BFGS, MLP regressor, ANN, RF, LG, CD-Net, FFNN, LASSO, DeepFM, ARIMA-WBF, LSTM, PODA

The data are divided into three groups, namely Multimedia Content, String Patterns and Time Series, the descriptions of which are provided on the top of the table. Next, for each group, typical sources and fields of application are reported with some explanatory examples. As for the application field we used the taxonomy presented in Chen et al. (2020). Finally, common AI techniques are reported for each column

**Fig. 2:**
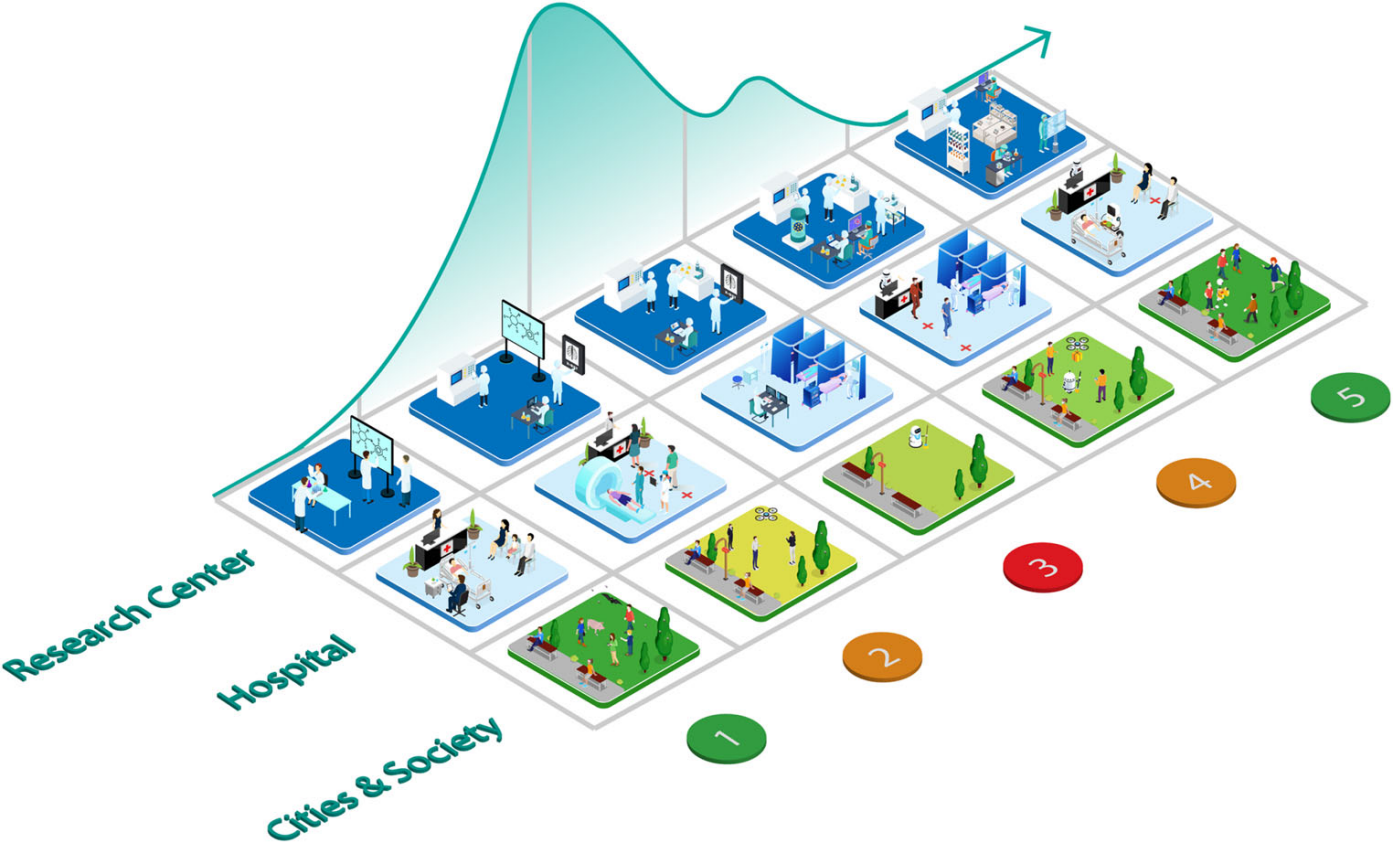
A time perspective of a pandemic diffusion, with advances in society, medical centers and research institutes. This Figure shows how the collection methodology on the type itself of data evolves in time and interwines with various areas of society. Clinical data generates health records, which, grouped in time and by population leads to time series, predicted by forecasting models to anticipate the epidemic evolution. Whereas X-Ray and CT generated in hospitals can be used to train DL models for diagnosis purposes. Starting from the bottom, the first stage represents the moment where a pandemic has not yet occurred. In society, there are no specific involvements, and in research institutes laboratories study and test new viruses and diseases. In the second stage, monitoring and social surveillance are performed to prevent any virus from becoming endemic and impacting on society before the availability of a full study on a cure or a vaccine. At the same time, hospitals are generating medical data on infected patients, like chest CTs as in this COVID-19 pandemic. Neural Networks (NN) can start to analyze such images, improving their accuracy in predicting new cases. The third stage is the occurrence of a pandemic, in which period, time series data might be showing an exponential trend. Here, the time series start to become longer, and predictions in time can be made with ML models. At the fourth stage, there is a significant number of cases and substantial amount of data, making necessary a text analysis of papers with the help of an AI tool in order to process the huge number of articles about the virus. Finally, when the pandemic is over, and a vast amount of data is available, a retrospective analysis can be carried out

Figure 2 illustrates the evolution in time of a pandemic while showing the different contexts and the related AI applications. The timeline reflects the sequence of decisions relating to how the different phases of the pandemic need to be addressed, according to the WHO GIP (2009). This institution identifies six pandemic phases plus two other periods (a post-peak and a post-pandemic). These can be grouped into five periods, according to the actions needed to be taken into account. The Fig. 2 shows data generated in different phases and areas of society. As the clinical data is generated from patients and analysed in laboratories, patient health records and time series are deduced from the pandemic data and X-Ray and CT are generated in hospitals; all this data results in a range of applications that AI can enhance: outbreak prediction, spread tracking, diagnosis, drug production and drug repurposing. Technically speaking, at different stages of the pandemic spreading timeline, scientific papers report information about the data collected and cases tested to investigate the disease’s characteristics.

In this work we also summarize the main research contributions related to the role of AI in the COVID-19 pandemic by reporting the Tables 2, 3, and 4 at the end of each subsection, in order to guide the reader through the principal literature results about application of AI in Covid-19 related contexts.

**Table 2:** Summary of COVID-19 outbreak (stage 2) related literature

Reference	Dataset	COVID19 Data	Time interval	AI/ML method	Performance	Relevance	Shortcoming
Randhawa et al. (2020)	National Center for Biotechnology Information (NCBI) database	**text**. Genome of 29,903 base pairs. 28 sequences of COVID-19 virus and the bat Betacoronavirus RaTG13	before Jan 23, 2020	Machine Learning with Digital Signal Processing (ML-DSP) approach, which uses 6 supervised learning approaches like Linear SVM and KNN, augmented by a decision tree approach to the machine learning component	Average ACC: 90.5 – 96.2	i) Confirm the taxonomy of the COVID-19, and possible bat origin; ii) alignment-free methodology adopted to rapidly analyze large datasets.	ML-DSP is a black-box method that does not offer a (biological) explanation for its output and is not able to assign a taxon that it has not been trained on.
Fong et al. (2020a)	Archive of Chinese health authorities	**time series**. 14 instances of suspected cases	Jan 21 – Feb 3, 2020	polynomial neural network with corrective feedback (PNN+cf)	RMSE: 136.55 RMSE Lin Regressor: 520.16 RMSE ARIMA: 1016.27	Data augmentation to the existing little data and fine-tuning the parameters of an individual forecasting model	Predicted result is very sensitive to the parameters used. Understand why algorithms incur very low or very high errors (for panel selection).
Fong et al. (2020c)	Chinese Center for Disease Control and Prevention	**time series**. 58010 of recent confirmed cases	Jan 25 – Feb 25, 2020	Broyden–Fletcher–Goldfarb–Shanno optimized polynomial neural network (BFGS-PNN), i.e. PNN enhanced with parameter optimization function.	RMSE: 62077.26 RMSE LinReg: 127693.55	i) BFGS algorithm to optimize the parameters and network structure size using alliteratively hill-climbing technique. ii) Estimate the direct cost that is needed as an urgent part of national budget planning to control the COVID-19 epidemic	Compare and contrast the differences of other techniques and refine input for accuracy.
Wang et al. (2020)	dataset of the radiology department of Huazhong University	**images**. Chest CT scans. 540 patients including 313 COVID-19 patients	Dec 13, 2019 – Feb 6, 2020	Weakly supervised deep learning framework (DeCoVNet): UNet (pre) & three stages 3D Deep Network & UNet	(ROC) AUC: 0.959	COVID-19 classification and lesion localization using 3D CT volumes	i) UNet model for lung segmentation did not utilize temporal information and it was trained using imperfect ground-truth masks ii) cross-center validations (more hospitals); iii) CT data of (CAP) not collected; iv) explainability
Kang et al. (2020)	Chinese CDC	**images**. Chest CT images. 2,522 patients including 1,495 COVID-19 patients	Jan 9 – Feb 14, 2020	FCNN: Structured Latent Multi-View Representation Learning	ACC: 95.5% Sens: 96.6%, Spec: 93.2%	Classify COVID-19 vs CAP. Use of multi-view representation learning with multiple features, such as texture, surface, volume histogram, and intensity.	i) Diagnosis with more classes instead of only two types of disease (i.e. COVID-19 and CAP). ii) Clinical characteristics of patients can be useful for diagnosis.
Xu et al. (2020)	2 China Hospitals	**images**. CT samples. 618 CT samples of which 219 came from 110 patients with COVID-19	Jan 19 – Feb 14, 2020	Residual network (ResNet)-18 by concatenating the location-attention mechanism in the full-connection layer to improve the overall accuracy	binary ACC : 86.7%	Multi-center case study. Location-attention classification model	Only compared the CT manifestation of COVID-19 with that of IAVP. To combine the patient’s contact history, travel history, first symptoms, and laboratory examination.
Mei et al. (2020)	18 medical centers in China	**images**. CT scan. 905 patients including 419 COVID-19 patients	Jan 17 – Mar 3, 2020	SVM, random forest and MLP classifiers	(ROC) AUC: 0.92 Sens: 84.3 Spec: 82.8	Compared performance to one fellowship-trained thoracic radiologist with 10 years of experience and one thoracic radiology fellow.	Explore various approaches, including 3D deep-learning models and develop the interpretability of CNN models. To validate the robustness of the models, is important to test the AI system in multiple centers.
Liang et al. (2020)	NHC of the People’s Republic of China	**time series**. baseline clinical features. 1,590 patients	before Jan, 2020	three-layer feedforward neural network & LASSO Cox model	C-index: 0.894 (ROC) AUC: 0.911	Deep Learning Survival Cox model had superior discriminating power compared with the classical Cox model, because it unravels the nonlinear relationships between complex clinical covariates and their hazards.	To extended deep learning model to integrate time-dependent covariates such as vital signs and high-dimensional features such a CT or X-ray images.
Wang et al. (2021a)	5 hospitals; most from hospitals in Wuhan, others from hospitals in Beijing.	**multimedia** CT images. 850 COVID-19 patients vs 541 non COVID-19	20 Feb 2020	i) classificaiton: ResNet-50, Inception networks, DPN-92, and Attention ResNet-50; ii) segmentation: fully convolutional networks (FCN-8s), U-Net, V-Net and 3D U-Net++.	best AUC: 0.991, with 3D Unet++ & ResNet-50	Experience in building and deploying an AI system	i) Does not perform well when there were multiple types of lesions, or with significant metal or motion artifacts, ii) The system is too dependent on fully annotated CT images.

The timings and data of the research papers analyzed are reported in the table. It can be observed that in this stage of the pandemic most of the works focus on the prediction of infection diffusion and early SARS-CoV-2 induced pneumonia diagnosis. Moreover, it is worth noticing that, since the first outbreaks occurred in China, a large number of the datasets used came from this country

**Table 3:** Summary of selected research papers about COVID-19 and data used within those studies

Reference	Dataset	COVID19 Data	Time interval	AI/ML method	Performance	Relevance	Shortcoming
Car et al. (2020)	JHU CSSE	**time series**. infected, recovered, and deceased patients. 20,706 data points for 406 locations	Jan 22 – Mar 12, 2020	MLP regressor. Limited-memory BFGS (Broyden–Fletcher–Goldfarb–Shanno algorithm)	*R*^2^ (confirmed): 0.94 *R*^2^(Recovered): 0.781 *R*^2^(Deceased): 0.986 on 5-cross validation	Model of novel viral infections with geographical and time data as inputs. Average training time 2357 min on 16 48-thread HPC nodes, for 5-fold cross-validation and grid search of 5376 items.	Models can be compared with various infectious diseases. Other approaches should be applied to gain explainability.
Zhu et al. (2020)	Huami Wearable Device	**time series**. Physiological data. 1.3 million users (with or without COVID-19)	Jul 1, 2017 – Apr 8, 2020	Regression model combining sparse categorical features and dense numerical features (CDNet), that concatenates 2 subnetworks: CatNN and DenNN	Pearson’s coefficient *ρ*: 0.68	Prediction using dynamic physiological data may have an advantage in recognition of the outbreak of infection.	The validity of the statistical description depends on both the user scale and diversity.
Ghamizi et al. (2020)	Google’s Mobility Reports	**time series**. 32 features (mobility trends over time and demographic features) for 97 different countries. 4,625 inputs of 32 features each	Jan 3 – Apr 29, 2020	Feed-Forward Neural Network (FFNN)	*R*^2^: 0.97 vs *R*^2^ (LSTM): 0.95	FFNN provides accurate and interpretable predictions	better feature engineering or neural architecture search (with CNN or RNN)
Mackey et al. (2020)	Twitter and Instagram	**text**. Sales of COVID-19 related products. 1,042 unique tweets and 596 Instagram posts	Feb 5 – May 7, 2020	NLP & RNN and LSTM	AUC: 94–99 (based on Li et al. (2019))	Identified over 1000 suspect selling posts	Multimodal methods that could analyze and distinguish both text and image have not been used.
Murphy et al. (2020)	Netherlands Hospitals	**images**. Chest X-rays. 994 images including 512 images from COVID-19 positive subjects	Mar 4 – Apr 6, 2020	CAD4COVID-Xray, based on CAD4TB v6 - a commercial deep learning system	AUC: 0.81 Spec: 78%; Sens: 75%	Performance compared against 6 independent readers	Need to take into account related patient details.
Ls et al. (2020)	2 hospitals in China	**images**. CT scans. 408 COVID-19 patients	Jan 1 – Mar 18, 2020	ResNet34 as a backbone model for multiple instance learning (MIL) framework training procedure	(ROC) AUC : 0.987 ACC: 97.4% on 5-fold cross-validation	Model can be employed as a tool for prognosis prediction. Validated a MIL-based predictive model using CT imaging.	i) Sample size was relatively small; ii)Lack of transparency and interpretability (like all DL models)
Zhang et al. (2020)	Wuhan and Ecuador centers; Radiopaedia dataset	**images**. CT images. 2,246 patients including 752 COVID-19 patients used for training	Jan 25 – Mar 25, 2020	(i) segmentation networks: U-net, DRUNET, FCN SegNet, and DeepLabv3. (ii) Classification networks: ResNet-18	ACC = 90.71% Sens = 92.50%, Spec = 90.00%	Performance comparable to that of practicing radiologists.	To refine the clinical prognostic model with varying risk thresholds associated with different clinical prognoses.
Abdel-Basset et al. (2021a)	Italian Society of Medical and Interventional Radiology	**images**. CT images. 80 COVID-19 patients used for image segmentation	before Apr 11, 2020	Few-shot segmentation (FSS) with four encoder blocks based on pre-trained Res2Net-50	DSC: 0.798 Sens: 0.803, Spec: 0.986	Model could outperform all approaches to multiple evaluation metrics	i) Comprehensive parameter improving to attain the highest results, ii) Predictions lack laborious uncertainty quantification, unable to achieve a very precise segmentation iii) Accountability and interpretability do need to be improved.
Roy et al. (2020)	ICLUS-DB	**video**. Lung ultrasound (LUS) videos. 35 patients (including 17 COVID-19 patients) generating 277 videos	Mar – Apr, 2020	ConvNet similar to van Sloun and Demi (2020), B-line, STN and CNN are jointly trained by using the Adam optimizer	ACC: 96% binary Dice score: 0.75	i) Fully-annotated dataset of LUS images, ii) Predicts the disease severity score associated with a input frame.	i) Leveraging the temporal structure between frames in a sequential model; ii) The data set should be wider and more balanced
Banerjee et al. (2020)	Hospital in Brazil	**time series**. laboratory test clinical data: age, outcome from SARS-CoV-2 test and standard full blood count (15 features). individual patients, including 81 COVID-19 patients	Mar 28 – Apr 3, 2020	i) ANN; ii) random forest (RF) and Lasso-elastic-net regularized generalized linear (glmnet); iii) simple logistic regression (LR)	(i) (ROC) AUC 0.95 ± 0.08 (ii) (ROC) AUC: 94% (iii) (ROC) AUC: 81%	Improve initial screening for patients with limited PCR-based diagnostic tools.	Random forests and glmnet offer a clearer overview of the most relevant factors, compared to ANN, as well as a better indicator on how a decision has been reached.
Pan et al. (2021)	2 isolation centers of Huazhong University of Science and Technology in Wuhan	**multimedia**. chest CT scans. 931 confirmed COVID-19 vs 1340 healthy persons	Until Mar 31, 2020	COVID-Lesion Net based on a combination of U-net and Fully convolutional networks	Dice coefficient: 82.08% 85.00% for the training	Deep learning-based quantification for COVID-19, quantification of the lung volume and the percent of the lung involvement.	i) Performance measured against no standard for the lesion area quantification for viral pneumonia, ii) Not multi-center training
Ismael and Şengür (2021)	three different sources (Cohen, Kaggle, Radiology Assistant)	**multimedia** Chest X-ray images. 180 COVID-19 and 200 normal (healthy) chest X-ray images	Mar 10, 2020	deep features model (ResNet50) and SVM with Linear kernel	94.7% accuracy other: 89.1%- 90.3%	Three CNN deep methods have been applied. In addition to different kernel functions, the deep features have been classified through SVM.	More testing needed.
Lopez-Rincon et al. (2021)	NCBI database of genetic variation and NGDC (National Genomics Data Center)	**sequences**. 583 sequences (*.fasta files) from the NGDC	Mar 15, 2020	CNN	Accuracy of 98.73	The network was able to systematically discover significant sequences to isolate the various virus classes.	Further testing is necessary

During the first peak of the COVID-19 pandemic (stage 3), the principal affected countries were in Europe and America and therefore the databases generally come from these areas. The first examples of social network analysis are reported, with a limited number of instances. The temporal windows during which the data were gathered extend until April 2020. Despite the time interval reported for Mackey et al. (2020), the relationship with Stage 3 for the COVID-19 is due to the fact that in the USA, by that time, the pandemic phase was still in the first stages

**Table 4:** Timing and data availability in research papers after the first peak of the COVID-19 pandemic (stage 4)

Reference	Dataset	COVID19 Data	Time interval	AI/ML method	Performance	Relevance	Shortcoming
Doanvo et al. (2020)	CORD-19 dataset (Lu Wang et al. 2020)	**text**. research paper collection. 48,670 COVID-19 papers vs 137,326 overall papers	before Jul 31, 2020	NLP & SVD & LDA	N.A.	ML explores latent semantic information to recognize hidden patterns and does not rely on any a priori knowledge of topics.	LDA is an unsupervised probabilistic algorithm and lacks the quality of a supervised method.
Ramchandani et al. (2020)	SafeGraph; Mapbox and The New York Times GitHub repository	**time series**. Features related to: population attributes, population activities, mobility, and disease spread. 2,100 sociodemographic features and others	Apr 5 – June 28, 2020	deep learning model based on the high-level framework of DeepFM	average ACC: 63.7%	Method can derive embeddings from multivariate time series and multivariate spatial time series data by using both the temporal and spatial structure in a wide range of input features.	No suitable method for interpreting second-order interactions; higher-order interactions are only indirectly captured and cannot therefore be easily interpreted.
Kim et al. (2020)	Google Search Trend; and datasets in *Data description* sec.	**time series**. Intra-country and inter-country time series. Daily cases and deaths with anxiety search trend; daily roaming entrants, airlines arriving, count of imported cases;	Mar 22 – May 5, 2020	Two-level hierarchical architecture of Hi-COVIDNet model, which mainly consists of the country-level encoder and the continent-level encoder.	RMSE: 0.4045 RMSE (ARIMA):0.4931 RMSE (multiv. LSTM): 0.5188	Exploit the geographic hierarchy as well as a hierarchical objective function to overcome a relatively short period of data collection for COVID-19.	Further testing is needed, on other country data.
Minaee et al. (2020)	COVID-19 Image Data Collection (Cohen et al. 2020b); Chestxray and ChexPert datasets	**images**. X-Ray images. 5,184 X-ray images including 184 COVID-19	before May 3, 2020	ResNet18, ResNet50, SqueezeNet, and DenseNet-161	Model Spec: ResNet18: 90.7*%* ± 1.1*%* ResNet50: 89.6*%* ± 1.1*%* SqueezeNet: 92.9*%* ± 0.9*%* Densenet-121: 75.1*%* ± 1.5*%*	Made 5k images dataset publicly available. Used transfer learning, and fine-tuning on pre-trained convolutional models	Only benchmark for future works and comparisons.It is worth noting that, considering the amount of data labeled, the outcome of the work is still preliminary and a more definitive conclusion needs more tests on a larger dataset of the COVID-19 labeled X-ray images.
Horry et al. (2020)	COVID-19 Image Data Collection (Cohen et al. 2020b); COVID-CT and POCOVID-Net datasets	**images**. a) chest X-Rays, b) CT scans and c) Ultrasound images. a) 729 patients including 139 COVID-19 patients; b) 746 patients including 349 COVID-19 patients; c) 911 patients including 339 COVID-19 patients	before May 11, 2020	Model Selection with VGG19 and others	F1 score: a)0.84-0.89, b) 0.81-0.83 c) 0.96-1.00	Provided a pre-processing pipeline aimed to remove the sampling bias and improve image quality. Showed pre-trained models tuned effectively for the Ultrasound image samples.	Needs great caution in the development of clinical diagnostic models using the available COVID-19 image dataset. To extend study with multimodal data fusion. A highly curated data set is not comparable to the available COVID-19 chest X-Ray dataset.
Jin et al. (2020)	3 centers in Wuhan; MosMedData, Tianchi-Alibaba, LIDC–IDRI databases	**images**. CT scans. 11,356 CT scans from 9,025 subjects of which 2,529 were COVID-19 scans	Feb 5 – Mar 29, 2020	i) Segmentation module based on U-Net, ii) Deep network backbone ResNet152, iii) Guided Grad-CAM for attentional regions.	AUC: 0.9745 - 0.9885 Dice (segmentation): 92.55%	AI system outperforms all of radiologists. Unlike classical black-box deep learning approaches, it can decode effective representation of COVID-19 on CT imaging.	Guided Grad-CAM can only extract attention region rather than lesion segmentation. It is important to collect more data and build a wide data set with linked CT and clinical information to allow additional diagnostic analysis.
Sadefo Kamdem et al. (2020)	Boursorama database; COVID-19 dataset	**time series**. Daily prices for 4 trading commodities; confirmed cases and total deaths in 2 countries	before Apr 24, 2020	ARIMA-WBF model and LSTM model	ACC: 92.13% - 97.45%	Forecasting commodity prices and examining the effect of coronavirus on commodity price fluctuations.	Application of reinforcement learning. Not analyzed price overreaction behavior.
Ou et al. (2020)	EIA weekly gasoline demand; datasets in *Data availability* sec.	**time series**. COVID-19 pandemic data, government policies and demographic information	Feb 15 – June 5 2020	PODA model, has 42 inputs, 2 layers, and 25 hidden nodes for each layer	RMSE: 6.2 - 65.2	Framework to investigate and project motor gasoline demand based on COVID-19 pandemic impacts.	Model does not consider the dynamic effect of travel mobility and assumes that the demand for gasoline from light-duty vehicles and other sectors is constant throughout the pandemic.
Wang et al. (2021b)	RSNA Pneumonia Detection Challenge & Cohen Dataset	**multimedia** chest X-ray. 3545 chest X-ray images, with 225 COVID-19	Jan 25 - May 1, 2020	ResNet50 + feature pyramid network (FPN)	accuracy of 95.12%	i) Automatically identify COVID-19 patients with X-rays. ii) Automatic lung detection. iii) Propose CAD tool for assisting in the processing of large-scale chest X-ray data	Lack of interpretability and not addressed disease classification by severity.
Gupta et al. (2021)	Five different virus diseases (i.e., Covid-19, Ebola, MERS, SARS, Swine flu)	**time series** Retrived number of affected cases and deaths from Coronavirus disease 2019 (COVID-19) time serie	Jan 22 - Oct 9, 2020	Dense layers with LSTM	RMSE: 0.0766-0.0533 RMSE (SVM): 0.2801-0.4323 RMSE (DT): 0.1108-0.1223	The proposed DL model has been compared to other popular prediction methods that indicate a lower RMSE.	For the model to work perfectly, the data must be accurate.

The time intervals generally extend from March to October, 2020. There is a wide variety of data sets, with different kinds of diagnostics images and social data. In particular, images derive from both CT and X-Rays, and many researchers use the Cohen et al. (2020b) X-Ray Dataset. In this stage data are freely available and are generated not only by hospitals: a wide variety is of social data can be found, e.g. encompassing gasoline demand Ou et al. (2020) and numbers of incoming visitors to a country Kim et al. (2020) . The different time positioning of Jin et al. (2020) concerning the Stage 4 of the proposed temporal subdivision, can be found in the fact that the authors refers to their country situation, the China, where the pandemic was already in an advanced state with respect to the rest of the world

Each table will present what in our opinion are the most notable papers for each pandemic phase. The fact that a paper belongs to a specific phase is based on the date in witch the data was analysed by the authors. The characteristics for each paper, contained in the tables at the end of the following subsections, can be divided into three macro areas: (i) Data related information, (ii) AI-related info, and finally (iii) pros and cons. As for the first area we report three columns, one about the dataset, the second about the type of data, concerning the categorization reported in Table 1, and the time interval in which the COVID-19 related data was collected. The second area reports a brief comment on the kind of ML or DL model used, or what could be considered more in general belonging to AI, and the performance obtained by the authors. These are reported for the classification task and for the regression task. As regards the former, we record either the Receiver Operating Characteristics Area Under the Curve (ROC-AUC), better known as AUC, that offers an overall performance metric indicating the chance that the model will correctly rate true positive and false negative occurrences; and also the Accuracy (ACC), which is the percentage of correct predicted occurrences (true positive and true negative). The motivation for the presence of this accuracy metric, although not ideal for imbalanced classes, is that sometimes it is the only measure reported on a paper.

As regards time-series prediction performance, we report the Root-Mean-Square Error (RMSE) which is a measure of how far from the model are the real data points. Finally, as for area (iii) we show what is the more relevant results for each paper moreover, limitations and drawbacks together with possible extensions to be done in further work is briefly discussed for each paper in the last column.

**Fig. 3:**
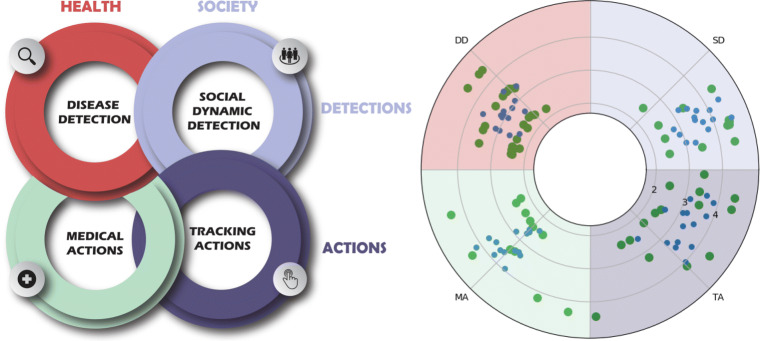
AI can be defined in terms of stages of observation and action. In the context of a pandemic, AI is applied in two main areas, namely medical research and the social context. Therefore, in order to study AI applied during a pandemic, we need to focus on four areas: disease detection (diagnosis), social dynamics observation (predictions), medical actions (treatments) and social management (tracing). Points plotted on the polar graph represent the papers in the literature. The positions deponent on how much each paper we analysed we considered belonging to each of area, within the 3 central stages of the pandemic

To better organize and explain all the possible applications during a pandemic of AI methodologies, we divided them into four main application areas: The areas shown in Fig. 3 encompass two pillars of AI - *action* and *detection* - related to two context, namely *society* and *health*. In each temporal stage, different pandemic dynamics act on the environment, determining consequences on social behavior and healthcare status. Reliable strategies to address dynamics issues have to be designed, and information about pandemic phenomenologies has to be collected and analyzed. In this perspective, a detection phase precides the actions phase. In the former, data are collected and organized, while in the latter, model outputs are used to produce an effect on the environment, e.g. simplifying a diagnostic procedure or developing a predictive strategy.

Figure 3 represents our before-mentioned framework. The figure is generated by assessing the confidence by which a paper can be assigned to a specific area. Then these papers are shown on a polar coordinate plot, where the radius interval indicates the temporal sage of the pandemic, and the angle assess the possible interconnections of a paper with respect to the other areas. The inter-annulus distance is used to deal with interconnections between opposite quadrants.

More in detail, each paper is identified by (*ρ*,*𝜃*), where *ρ* = *i* + *r*, where *i* ∈ [2,3,4] is the discrete value indicating the pandemic stage on the time line, *r* ∈ (0,0.5) asses the confidence of belonging to that quadrant or to the opposite one. While $\theta = q \pi /2 - \pi /4 +\theta ^{\prime } \pi /4 $ is built in such a way that *q* ∈ [1,2,3,4] fixes the middle of each quadrant, and $\theta ^{\prime }\in (-1,1)$ indicates the confidence of that paper belonging to that quadrant $\theta ^{\prime }=0$ or to one of the contiguous quadrants.

By reading from the upper-right quadrant and going clockwise, the reported papers mostly discuss issues related to social dynamics detection (SD), giving insights into what actions could be taken during the pandemic . In sector TA (where there is the least number of papers), they tended to express the suggested TA through medical information at the beginning phases. Furthermore, by considering sector MA, most papers in the first phases focused on the actions to involve for COVID, based on the data of the COVID-19, while in the last phase of the pandemic, those actions also intertwined with social dependent-actions. Finally, as the pandemic progressed, the last area, DD, got more focused on proposing specific tools for COVID-19 detection.

Considering the above premises, in the following sections, we discuss where AI may intervene during the different periods of a pandemic, focusing on what has happened during the COVID-19 emergency. The main implications for society and healthcare systems are examined for each of the pandemic phases in order to try to understand how this scientific research field relates to the difficulties and necessities that arise in a pandemic crisis.

### Before a Pandemic

Can the incidence of a pandemic be avoided? It is impossible to give a definitive answer to this question, but we can discuss the tools and the methodologies to be put in place to prevent the widespread diffusion of an epidemic. An infectious disease occurs when a pathogen contaminates one person transmitted from another person or, sometimes, when it “spills over” from its original host to a new host belonging to another species. The latter is the presumed origin of Sars-CoV-2, the COVID-19 causing virus, which has probably jumped from *bats* to humans via an intermediary mammal (Chellasamy et al. 2020). According to phases 1-3 of the WHO procedure, to which this first analyzed period corresponds, preventing such a situation depends on a healthcare organization ready to scale up and on efficient animal health monitoring systems. In this context, infectious disease monitoring is an essential and comprehensive process in which information on infection outbreaks and vectors is continuously and systematically collected, analyzed, and interpreted (Chae et al. 2018).

Monitoring strategies have to be flanked by centralized and easily accessible databases encompassing epidemiological, clinical and genetic data to elaborate strategies optimizing hospital operations, diagnosis, prevention, and therapeutics (Alimadadi et al. 2020). Following the outbreak of avian H5N1 influenza, more than 70 scientists signed a letter, Bogner et al. (2006), urging the creation of the Global Avian Flu Data Sharing Initiative (GISAID), thereby making it possible to achieve global access to new virus sequence data. These databases, immediately updated and continuously monitored, give researchers the ability to prepare for the next outbreak. A notable example of effectiveness in exploiting different and well-organized data sources is provided by the Canadian company BlueDot, which achieved a timely recognition of the COVID-19 infection by using AI and its ability to review more than 100 datasets contemporaneously. In Wuhan, China, on December 31st, 2019, BlueDot highlighted as significant what was then considered only an outbreak of pneumonia and predicted the likely course of the disease diffusion (Bowles 2020). Once data are collected, AI algorithms can be used as an initial screening tool for suspected infection cases. Patients with a higher risk can receive confirmatory laboratory-based tests or be immediately isolated (Ting et al. 2020), and molecular diagnostics for drug response can start at the first stage of a possible pandemic (Esteva et al. 2019). Furthermore, thanks to the virus’s genetic sequencing, the earliest possible data for investigation on drug repurposing is already available before any kind of outbreak (Ekins et al. 2019).

Implementing daily routines for a fast analysis of clinical data, such as automatic CT diagnosis, can support the healthcare systems. Furthermore, implementing monitoring strategies on different layers of society can afford a non-invasive procedure in the early recognition of a potential epidemic situation. For instance, SENTINEL from ęerban et al. (2019), is an application for syndromic surveillance through social media analytics, providing early warning detection and situational awareness by intaking data from multiple sources. This framework, developed with open-source software, uses a DL methodology to classify tweets and news articles related to health symptoms.

Detection strategies focused on infectious animal disease contexts can prevent any jump between species and the possible diffusion of a viral infection. Examples in this field are the automated reporting and monitoring service of mosquito vectors, which can be used to keep track of many potential vectors of serious diseases and pathogens; like malaria, West Nile virus, Zika virus, chikungunya, yellow fever, dengue, lymphatic filariasis and many forms of encephalitis (Fanioudakis et al. 2018). Besides, DL methodologies for the early detection of respiratory diseases in growing pigs based on environmental sensor data are presented in Cowton et al. (2018).

Considering the cited literature, AI could have a significant impact in preventing the outbreak of a pandemic by adopting methodologies and general-purpose tools able to adapt themselves to critical scenarios quickly. Infectious disease monitoring, drug repurposing, fast and accurate data analysis, social media analytics and learning methodologies for the early detection of human diseases are topics where AI could play a crucial role.

### At the Verge of a Pandemic

At this stage, influenza outbreaks will have been confirmed in several areas of the globe, and pandemic risk is increasing. According to the WHO guidelines, countries where the infection is spreading should apply containment strategies, while regions not affected should be ready for an immediate response. This phase is characterized by limited knowledge of the novel epidemic. The high uncertainty and complex social-political dynamics make it challenging to realize reliable predictions and design accurate control strategies. Apart from public datasets dealing with similar problems, not much data are available.

A brief summary of the literature explored about this stage can be found in Table 2.

In the specific case of the COVID-19 pandemic, the first accessible scientific information was the SARS-CoV-2 whole genome sequence, used to identify potential drugs for its treatment (Beck et al. 2020). Based on these data, the detection of medicine candidates and possible drug combinations targeting the COVID-19 virus has been explored through drug repurposing approaches (Zhou et al. 2020a, 2020b) to reduce the time and lower the cost in comparison with ex-Novo drug discovery. At the same time, thanks to AI applications, techniques have been developed to gain a more in-depth understanding of the pathogen: for instance, methodologies that can provide a rapid classification of novel viruses (Randhawa et al. 2020), identifying their intrinsic genomic signatures, can be used to recognize similarities with other known pathogens.

Once the basic mechanisms of transmission have been identified, Deep Learning techniques can be applied as support tools in containing the infection. In particular, as regards human behaviour control, which is not strictly linked to a specific pandemic, pre-existing applications can be reused for mask usage monitoring and distance recognition (Balakreshnan et al. 2020), or to identify people who are coughing by using data collected with drones or security cameras (Ramadass and Arunachalam 2020). As an example, Loey et al. (2021) proposed a hybrid model for face mask detection using deep transfer learning and classic ML classifiers. Simultaneously, with the epidemic rapidly spreading and the effect of restrictive measures becoming measurable, information about the temporal growth of the infection is now available. Data augmentation techniques and forecasting models can be applied to obtain predictions on the spectrum of possible scenarios (Fong et al. 2020a; Fong et al. 2020c) to increase the reliability of planning activities concerning new containment measures and the organization of healthcare structures.

Hospitals and clinics are becoming increasingly involved in the outbreak response, and intelligent technologies are increasingly important to achieve quicker diagnosis and enhance functionalities in medical practice. In the case of the COVID-19 pandemic, diagnostic AI tools, particularly in image recognition, have been investigated. Researchers have created models that automatically distinguish the pneumonia caused by the SARS-CoV-2 virus from other lung diseases, using data specifically obtained at their hospitals mostly collected from China (see Table 2) (Wang et al. 2021a; Wang et al. 2020; Kang et al. 2020; Li et al. 2020). A remarkable example is proposed by Xu et al. (2020): they have developed an early screening DL-based model able to differentiate COVID-19 pneumonia from influenza-A pneumonia (IAVP) and healthy cases via pulmonary CT images. This technique has been proved to be a promising back-up diagnostic tool for frontline clinical doctors. Moreover, the availability of different data types allows enhanced performances through information-fusion practices, such as combining CTs with clinical symptoms, the history of exposure, and laboratory tests (Mei et al. 2020).

Abdel-Basset et al. (2020) propose a hybrid approach to overcome the Image Segmentation Problem for COVID-19 chest X-ray images based on the integration of meta-heuristic strategies to maximize the Kapur’s entropy.

When the number of critical cases explodes exponentially, additional issues arise: it becomes essential to keep non-infected patients separated from infected ones to prevent new outbreaks, especially in healthcare facilities attended by physicians. Consequently, efficient early triage procedures must be implemented, for example, by using AI to predict the likelihood of critical disease development in COVID-19 patients based on their clinical features at entry (Liang et al. 2020). Besides, the management of Intensive Care Units (ICUs) constitutes another crucial task: there starts to be a great number of critical patients, and, by considering the deficiency of doctors and equipment, efficient methods of organizing personnel and therapies are required. To aid clinical decisions, models for patient management that leverage IoT devices capable of gathering physiological data from ventilators and other medical devices have been developed during the COVID-19 emergency (Rehm et al. 2020).

As for vaccine development, Crooke et al. (2020) were the first to propose a ML workflow (based on a combination of a hidden Markov model and a propensity scale method) for analyzing the SARS-CoV-2 proteome, available by February 27,2020, and identify possible T-cell and B-cell epitopes, that could serve for the production of peptide-based vaccines.

The incidence of the COVID-19 pandemic has taught us that there is limited knowledge of novel diseases. AI provides a good example of how support can be given in planning effective strategies in healthcare emergencies. In detail, in the medical context, developing methods for an in-depth understanding of the pathogen and learning-based models able to recognize COVID-19 pneumonia are important research fields to investigate. Concerning society in general, tools for mask usage monitoring and distance recognition and forecasting models to obtain predictions about possible future scenarios and the occupancy of medical facilities are topics to which AI could have a significant impact.

### The Pandemic Begins: to the First Peak

On 11th March 2020, the WHO Director Dr Ghebreyesus used for the first time the word *pandemic* to characterize the outbreak of COVID-19. Countries such as Italy, Iran, South Korea and Japan had registered growing numbers of cases by that time, with the virus spreading to over 100 countries and infecting more than 120,000 people (McNeil 2020). Several collaborative efforts on society and medical and research centres were needed to deal with this global issue. In Table 3 the reader can find an outline of the discussed papers in this subsection.

One of the main issues in this phase is to minimize the pandemic’s impact on healthcare systems. The importance of containment measures becomes central to prevent catastrophic situations, and tools to enhance estimations of the number of infected people in specific geographical locations can allow wise planning of ICUs and emergency structures (Car et al. 2020). In these different contexts, methodologies based on AI models start to be developed to support the quarantine verification task. As a first attempt, AI can support video-surveillance systems to check the correct usage of masks or enhance public health monitoring through IoT data (Zhu et al. 2020). Models able to identify irregular patterns and early signs of the outbreak of the pandemic, as in Karadayi et al. (2020), are also developed to design strategies to prevent the collapse of healthcare facilities (Car et al. 2020). Furthermore, simulation toolkits, providing estimates of the epidemiological parameters combined with components seeking the optimal trade-off policies between the decision makers’ constraints and goals (Ghamizi et al. 2020), evidence how AI models can be used to model problems connected with the spread and impact of infectious diseases based on geographical and time data as inputs.

The first diffusion of a pandemic in a global hyper-connected world has not only opened up new forms of international collaboration but has also revealed novel challenges to face. Misinformation and fake news, fraudulent sales of suspicious immunity-boosting treatments and health-related goods, and not approved medical treatments have highlighted the need for new policies and systems able to collect, evaluate, recognize and report dangerous news and counterfeit products to address these new social and economic issues (Mackey et al. 2020). Healthcare structures are now under stress, and, especially in hospitals, tasks such as the optimization of diagnostic procedures and the isolation of infected patients reach their peak importance.

Concerning COVID-19, the SARS-CoV-2 virus has been found to attack most frequently the lungs, causing pneumonia-related issues; therefore, the principal diagnostic tool consists of medical imaging for this kind of disease. In this context, AI has found widespread applicability in the diagnostic task due to the well-known capabilities of, in particular, DL models to work in the field of image segmentation and recognition. Thanks to data collected during the earlier phases and to the collaborative effort of many scientists worldwide, some previously conjectured strategies can now be applied in real contexts. As regards AI, the huge number of medical images collected has, for example, enabled medical practitioners to exploit X-ray images (Dhiman et al. 2021). These examinations are more economical and expeditious with respect to CTs to diagnose the COVID-19 disease; here, transfer-learning techniques can be exploited. The CAD4COVID-XRay tool (Murphy et al. 2020), developed by Thirona (Nijmegen, the Netherlands) and firstly used to aid in the tuberculosis diagnosis from CXR images, is now trained on chest X-ray images to detect COVID-19 related pneumonia. Such AI system performances in the detection of COVID-19 were found to be comparable to those of six independent domain experts. In addition, a recent work from Ismael and Şengür (2021) applied a specific DL technique, the ResNet50, on 180 COVID-19 X-ray images. Moreover, thanks to the availability of X-ray in healthcare facilities, these systems can be integrated with CT scanners, both of which are commonly used in frontline hospitals (Shi et al. 2020). The wider availability of CT has done possible intensive research in this area by increasing the data in input and the quality of the predictions and allowing AI systems to be openly available to practitioners worldwide (Ls et al. 2020; Zhang et al. 2020). A semi-supervised few-shot segmentation (FSS) approach of 2019-nCov infection from a few amounts of annotated lung CT scans was developed by Abdel-Basset et al. (2021a), their approach leads to overcome the necessity of huge dataset in DL Networks training phases. They reached a Dice similarity coefficient (DSC) of 0.798. Pan et al. (2021) developed COVID-Lesion Net based on a combination of U-net and Fully convolutional networks.

In addition to the measured accuracy of the applied strategies, in this phase of a pandemic, the diagnosis’s timing is also essential. Early diagnoses allow efficient planning of patient sorting, reducing the pressure on medical structures, and managing hospitalizations. In this context, AI can be applied to rapid examinations, such as lung ultrasonography (LUS) images (Roy et al. 2020) or blood counts (Banerjee et al. 2020), to obtain an estimation of the degree of severity of the disease, and thereby to organize more efficiently admissions to ICUs.

Treating patients, however, renders the hospital personnel vulnerable to the infection, and so brings the risk of reducing the capability of the healthcare system facing the pandemic emergency. Essential tasks, such as environment disinfection, expose workers to contaminated sites, increasing the possibility of a further diffusion of the virus. However, thanks to AI, a partial solution can be found in robotics, whose utilization has been explored during the COVID-19 crisis and sometimes adopted. It is the case of a mobile robot system for personal care able to perform support functions such as the disinfection of premises and monitoring body temperature (Miseikis et al. ). Applications of such robotic solutions, autonomous and/or remote-controlled, about cleaning tasks mainly have proven to be cost-effective and secure (Ramalingam et al. 2020) so representing a different tool that AI can put in place to fight such a situation of global emergency.

Finding strategy to prevent reduce the impact of the epidemic on the society is now compelling: investigation on possible vaccines through techniques of drug discovery and repurposing becomes a more and more important topic in the research. Malone et al. (2020) applied Monte Carlo-based simulation to forecast blueprinting for SARS-CoV-2 vaccines, by providing a wide range of T-cell epitopes (Antigen part that can stimulate an immune response). The Immunitrack ApS authors, Prachar et al. (2020), a company that provides a immunogenicity assessments during drug development, used the neural network method, NetMHC, to predict which peptides will bind, and so identify epitopes for SARS-CoV-2 vaccine. Finally, as for analyzing COVID-19 gene sequences, Lopez-Rincon et al. (2021) propose a CNN to classify 553 genome sequences with promising accuracy results.

### After the First Peak of the Pandemic

At this stage, thanks to restrictive measures and adequate surveillance, the diffusion of the virus is contained, and the number of new infections starts to decrease. However, since the level of pandemic influenza activity might increase again, evaluating the response of social and healthcare systems and analyzing critical issues raised in earlier phases become essential tasks to avoid a potential new wave of contagion. According to the WHO, it is necessary to determine the efficacy of the interventions employed to date and possibly to update guidelines, procedures, and strategies. In this sense, undertaking retrospective studies helps minimize the impact of the pandemic and decrease the risk of other outbreaks.

The paper concerning this stage are resumed in Table 4.

Data is constantly flowing, and research communities start to share information in a systematic and structured way: the COVID-19 Drug and Gene Set Library Kuleshov et al. (2020), in which a collection of drugs and gene sets related to research into COVID-19 from multiple sources is present. The COVID-19 Open Research Dataset (CORD-19) Kohlmeier et al. (2020), containing weekly updated research papers on COVID-19, SARS-CoV-2 and related coronaviruses, are just two remarkable examples of collaborative platforms put in place during the current situation. In this scenario, such a publicly accessible knowledge collection expands research possibilities and makes AI methodologies increasingly feasible.

Natural Language Processing methodologies can be used to quickly analyze the scientific results produced in literature (Doanvo et al. 2020; Kricka et al. 2020; Levin et al. 2020). In particular Doanvo et al. (2020) used NLP to lemmatize words and preprocess text, and achieved dimensionality reduction with SVD, and topic modeling with its LDA. Moreover, DL techniques can be applied in the understanding of social and political aspects through the press and social media information. Besides, temporal trends can be studied and analyzed thanks to ML and DL forecasting models, and mathematical models, such as Susceptible, Infectious, or Recovered (SIR) models, can be extended and enhanced thanks to AI strategies.

The diffusion of human infections can be kept under control by social distancing and isolation practices. This simple application of common sense (as social distances), as in the case of coronaviruses Chatterjee et al. (2020), comes at a high price in terms of its social and economic impact. It is clear that smart strategies for detecting infected individuals and monitoring their behaviours constitute a fundamental precondition to relax the restrictive measures necessary at the pandemic’s peak. AI can provide tools able to recognize early symptoms, such as fever in the case of COVID-19, through its applicability in computer vision tasks. For example, automated measurement of the body temperature at different checkpoints in airports, train stations, and various city locations is presented in Barabas et al. (2020). Thanks to cameras’ pervasive presence for surveillance and traffic detection, it is possible to identify and track people in close contact with suspected COVID-positive individuals. Such strategies can allow the early identification of potential outbreaks, avoiding new contagion waves thanks to localized quarantines for people who may have been infected. In principle, compliance with confinement can be assured through passive monitoring and active verification, thanks to smart home devices nowadays widespread. For example, an interesting application involves smart speakers (Alrumayh and Tan 2020), which regularly listen in an environment and only transmit a signal when the local monitoring algorithm senses that someone is not at home.

From a more general perspective, monitoring strategies, although valuable allies in the task of keeping potentially catastrophic outbreaks under control, cannot be ubiquitous. Resources are limited, and monitoring systems cannot be placed everywhere. Methodologies able to forecast the temporal behaviours of the infection, given population attributes, population behaviour, mobility, and disease diffusion features, Ramchandani et al. (2020) can help in identifying high-risk situations in advance. Models for mobility prediction can be used to understand geographical epidemic relations and the risk of contagion. A remarkable example is the two-level components neural network, Hi-COVIDNet, developed by Kim et al. (2020), consisting of country-level and continent-level encoders, that allows an evaluation of the hazard of international displacements. In this sense, the strategies mentioned above can support governments in allocating quarantine resources and can also optimize both the efficiency of any restrictive measures imposed and the arrangement of monitoring systems.

Enhanced early diagnosis procedures, in a sense, constitute another critical prerequisite for less restrictive social isolation measures. Enhancing the methodologies that allow a rapid and accurate diagnosis of the infection from symptoms and medical analysis ensures that patients can be sorted in an increasingly efficient way, resulting in more effective treatments. Such an approach produces benefits both for people and healthcare systems. An improved ability to manage the illness enhances the robustness of the response to possible new emergencies. We highlight results from Gupta et al. (2021) that study the trend of five different virus diseases (Covid-19, Ebola, MERS, SARS, Swine flu) by using LSTM. The medical data collected during the peak of the emergency and information about the applicability of different models allow further refinement of the procedures mentioned above, focusing the research on practices that have proven to be effective.

The literature reviewed shows that two main approaches can be followed to accomplish this task. A first strategy consists in exploiting pre-trained convolutional models, fine-tuning them on COVID-19 related diagnostic images (Minaee et al. 2020; Horry et al. 2020; Wang et al. 2021b). In this way, it has been possible to obtain estimates of lung regions potentially infected by COVID- 19, subsequently examined with certified radiologists’ help. Secondly, research has focused on one of the most challenging problems emerging with the advent of seasonal influenza, namely differentiating between SARS-COV-2 induced pneumonia and common pneumonia. It is worth highlighting that separating COVID-19 patients from those not infected ones is a critical task, especially in healthcare structures, since an already compromised patient can have severe implications for her/his health. With this aim, new AI approaches have been developed. Among these, we report only a few examples, such as Jin et al. (2020), who proposed an AI system for rapid COVID-19 detection, developing and evaluating this on a large dataset with more than 10,000 CT images from COVID-19, influenza-A/B, non-viral community-acquired pneumonia (CAP) and non-pneumonia subjects, and Oh et al. (2020), who using public chest X-ray (CXR) datasets, investigated potential biomarkers in CXRs and exploited their findings to develop a patch-based deep NN architecture that can be stably trained with a small dataset.

After fighting the pandemic through monitoring, distancing and enhanced medical procedures, once the emergency peak has passed, the effects of the crisis on the community have to be faced. The forced closure of commercial activities and the extended isolation have generated economic and social issues, drawing institutions’ attention to financial support plans concerning the former problems and community-related prevention strategies about the latter. In this context, AI strategies can prove valuable in optimizing resources and designing tools for efficient analysis: predicting the temporal evolution of different market segments (Sadefo Kamdem et al. 2020; Polyzos et al. 2020; Ou et al. 2020) can result in more efficient strategies for the redistribution of funds. In contrast, the predictive analysis of mental health disorders due to high distress during the COVID-19 pandemic (Ćosić et al. 2020), for example, can lead to the establishment of social services infrastructures where they are most needed.

### After the Pandemic

This final state is defined by WHO GIP (2009) as the *post pandemic* phase. It is the case the virus presence could be endemic in the population, and viral occurrences are comparable to the ones of seasonal influenza in most countries with appropriate monitoring. In this stage, all the strategies put in place during the different phases of the pandemic have paid off, and the infection levels are under control.

In 2015, Gates (2015), after the Ebola epidemic, pointed out that the world needed global warning and response mechanisms to prepare more effectively for possible future pandemics. Concerning the COVID-19 pandemic, the world was still not ready to face such a challenge, and indeed this event may serve as a lesson for governments to implement the kind of mechanisms Gates was proposing. In this context, a retrospective analysis can highlight the measures that worked well and those that did not provide useful guidelines about the right strategies to put in place during a health crisis.

From a data perspective, the great efforts made by research communities in terms of providing shared and open-access databases have facilitated international collaborations (e.g. the 4CE international consortium for Electronic Health Records (EHR) data-driven studies of the COVID-19 pandemic (Brat et al. 2020) or the COVID-19 related projects from the ELLIS network, see https://ellis.eu/covid-19/projects for more details). This kind of initiative enhances coordination, reducing the risk of unnecessary repetitions of similar research studies.

Moreover, the availability of such information supports the design of AI methodologies to integrate different types of data. Clinical symptoms and laboratory analyses will lead to reliable and effective diagnoses (Shi et al. 2020); the availability of a multitude of “big data” sources about human-to-human interactions will provide an efficient tracking of the infection (van der Schaar et al. 2020), and an improved decision-making process.

Other examples include aggregate AI models to develop Clinical Decision Support Systems (CDSS) systems for ICUs, exploiting IoT systems (Rehm et al. 2020). Also, the use of AI in the field of robotics to explore the cost-effectiveness and the benefits of such strategies applied in pre-, intra- and post-operative care situations can be considered (Zemmar et al. 2020). The application of transfer learning to account for differences between populations, eliminating biases while still extracting useful information to identify appropriate subgroups, is used to speed up learning phases in testing new treatments (van der Schaar et al. 2020).

Furthermore, concerning the modelling of knowledge, Reese et al. (2021) analyzed biomedical data from 15 Mar 2020 to 8 Jan 2021 to produce knowledge graphs (KGs) with a framework that ingests and integrates heterogeneous data. The framework uses *node2vec* to obtain graph embeddings for generating predictions. This approach may provide a way to merge different data sources and knowledge and form a platform to facilitate COVID-19 response. On the other hand, Laponogov et al. (2021) used databases of food molecules and drugs to model a random walk propagation algorithm to propose drug/food-based compounds with the highest probability of being effective against COVID-19. They propose a ML method to identify potential bioactive anti-COVID-19 molecules in foods based on their ability to target the SARS-CoV-2-host interactome networks. The authors conducted their research within the supercomputing DreamLab App network, utilizing thousands of smartphones’ idle computational capacity. The authors also expect this food map project to play an essential role in the future of precision nutrition therapies against COVID-19.

Ultimately, from a general perspective, the authors in Abdel-Basset et al. (2021b) describe and discuss a combination of different technologies for COVID-19 analysis. Such technologies fusion include AI, industry 4.0, IoT, Internet of Medical Things (IoMT), big data, virtual reality (VR), Drone technology, Autonomous Robots, 5G, and blockchain to deliver jointly innovative solutions.

The huge boost in AI-related research and all the strategies so far discussed emphasise that these kinds of approaches can offer effective solutions to real problems and can be pervasively applied in many different fields. Under the ubiquitous presence of IoT devices and new connection technologies, acknowledging this fact opens the way to a multitude of new business opportunities. As an example, due to the mass use of wearable devices, the Fitbit company is working with Apple company, and the Stanford Healthcare Innovation Lab on algorithms that can detect COVID-19 even before any symptoms have started (Snider 2020). Mila, is another exciting example, which brings its DL expertise to the scientific world, together with its partners across various disciplines, to find and implement solutions to pandemic related problems (see https://mila.quebec/en/covid-19/).

## Discussion: Where we are and What is Next

The proposed overview of scientific works published during the ongoing COVID-19 pandemic highlights some achievements and limitations of AI in tackling the 2020 pandemic. By considering the four domains mentioned in Fig. 3 (health and society applications able to detect and act), there are still requirements that AI cannot quite attempt. Indeed, AI still holds three well-known features which can result in potential failure: the absence of strong AI, its inability to work without knowledge of the domain, and the need for good quality and flow of data. Furthermore, ML and DL techniques must be scrutinized in order to determine what are the best current solutions, as well as future developments and research perspectives, without ignoring ethical concerns (such as trust and privacy) that currently hinder AI usage in our society.

In the previous sections, we have analyzed the pandemic concerning five-time stages, each of them explained using four application areas. In each of these areas, AI has already given a significant contribution to society and healthcare, but considerable development is still required. This section will first discuss examples regarding the four areas of Fig. 3; it will then consider machine learning techniques related to the data types of Table 1, and trends in ML/DL will be discussed. Finally common issues related to AI will be analysed.

### Fields of Application

We present and discuss an analysis of the limitations associated with this topic with specific reference to each of the four areas mentioned in Fig. 3.

Regarding population control, there is still a gap for AI solutions to fill concerning contact tracing and distancing techniques. In contact tracing, AI’s role seems to be anything but central since no AI features in contact tracing apps appears to be deployed. Additionally, applications for social distancing regulation, or even for the replacement of humans in labour-intensive contexts, seem to exclude AI. The case of Taiwan can represent an exception since ML has helped the Taiwanese government undertake contact tracing. They rated the population as either at a lower or higher risk based on different variables (e.g., travel history); then, they distributed mobile phones to infected people to track their GPS location. In this way, it was possible to enable the police to monitor infected people movements ensuring that they do not move out of their confinement area Cohen et al. (2020a). However, the Bluetooth signal detection technology that can identify nearby phones seems very inaccurate. There are challenges to overcome before that ML can effectively boost Bluetooth-based proximity detection by integrating similar data from other phone sensors gyroscopes and accelerometers Hsu (2020).

Regarding population monitoring, AI applications can still be viewed at an embryonic stage in studying the predictability of a pandemic. Outbreak in Wuhan was claimed to be predicted by BlueDot (Fong et al. 2020b) with AI and ML; however, the existing scientific literature lacks in showing consistent AI advantages over traditional methods.

Heinson et al. (2017) used SVM to predict antigens in a Reverse vaccine (RV) problem. About the research for vaccines and COVID-19 treatments, the use of AI is gaining more attention, also thanks to international projects as CoronaDB-AI, a data set containing genomic features, that can be used to train AI models for COVID-19 treatments (Keshavarzi Arshadi et al. 2020; Liu et al. 2021). Recent researches (Crooke et al. 2020; Malone et al. 2020; Prachar et al. 2020) uses Monte Carlo-based simulation, hidden Markov model or neural network methods to predict epitopes, the antigen part that can stimulate an immune response, as possible targets in a vaccine development. Notably, Yang et al. (2021) proposed a deep learning approach (DeepVacPred) for prediction and design of a multi-epitope vaccine capable of predicting 26 possible SARS-CoV-2-spike-protein sequence vaccine subunits. Moreover, Ong et al. (2020) propose a solution is based on hidden Markov model (HMM) and a neuronal network architecture known as SPAAN, which uses the reverse vaccinology technique for predicting COVID-19 vaccine candidates. Unfortunately, for the research and development of vaccines and anti-COVID treatments, it appears that only few researches has made extensive use of AI solutions. In our opinion as far as publicly available, AI still appears to not be widespread applied in the academic literature vaccine research. Conversely, a good example is provided by the American company Moderna, which has managed to reduce the time needed to produce a human-testable prototype of vaccine by making use of bioinformatics solutions in which AI seems to have played a pivotal role, as reported in the Science magazine by Cohen (2020).

On the other hand, there are many positive examples in diagnostics, particularly in imaging, where effective research has been carried out, and concrete applications developed even in the corporate world, such as REiLI, Fujifilm’s Artificial Intelligence platform (Porfido 2020). Concerning diagnostics, the real limitation seems to be the resistance of physicians to trust AI. Therefore, a specific effort would be needed to incorporate new technologies into an existing professional context in an effective way (Elish and Watkins 2020).

### Machine Learning Aspects

ML and DL methods are usually dependant on the application domain and on the kind of data. As summarized in Table 1, it is possible to identify three main kind of data that driven the applications: multimedia, string, and time-series data. In some areas, DL/ML has shown to be applied successfully, but, at the same time, more traditional techniques still are used. In the following, we discuss each of the following areas connected to the aforementioned data types: (i) computer vision processing, (ii) omics and text analysis, (iii) predictive analytics.

Regarding (i), the key tasks required in the field of multimedia content analysis are either segmentation or classification. The type of data analyzed during the COVID-19 pandemics includes video images of Chest CT, Chest X-Ray (CRX) and lung ultrasonography (LUS), and other applications, including audio cough recognition, image mask detection.

As for classification process, DL architectures are widely used, e.g. Horry et al. (2020) compared VGG16/VGG19, Resnet50, Inception V3, Xception, InceptionResNet, DenseNet, and NASNetLarge, and other comparison have involved ResNet18, ResNet50, SqueezeNet, and DenseNet-161 (Minaee et al. 2020). In general the most common DL method is the residual neural network ResNet with its various modification, extensions and parameter tuning.

By considering chest segmentation in medical domain, this task is usually performed for CT scan, given its 3D nature, rather than chest X-Ray (CRX): in the literature research many segmentation techniques are applied on CT scans, since the need for tools which help in extracting meaningful insights are more compelling, to practitioner, due to the complexity of 3D visual reconstructions. Full convolution networks (FCN-8s), U-Net and 3D U-Net++ (Cao and Bao 2020; Wang et al. 2021a) are commonly used as segmentation models. A final technique that appears to be promising in the field of medical image classification is the Gradient-weighted Class Activation Mapping (Grad-CAM) on CNNs: this technique offers interpretable outcomes proposing, to practitioners, attention regions to focus on Jin et al. (2020).

With respect to (ii), two major application domains can be identified in the field of string patterns: drug repurposing or drug exploration to classify DNA sequences with particular functions and applications, and text mining for reviewing COVID-19 related literature. Natural language processing (NLP) is used to transform unstructured text into normalized suitable structured material. SVD is typically used in the field of text analysis techniques. LDA, an unsupervised probabilistic algorithm, is used for modelling topic tasks. In drug repurposing, to identify associations and forecast drug-disease interactions, text mining techniques and graph-based advisory systems are used. In predicting drug likeliness, drug target relationship, and generation of novel molecules against the desired target, autoencoder approaches help. More DL like examples is Graph Convolutional Network with Attentional mechanism for Drug–Disease Interaction (Att-GCN-DDI) as in Che et al. (2021), and Molecule Transformer-Drug Target Interaction (MT-DTI) to predict any commercially available antiviral drugs that could be effective against SARS-CoV-2 (Beck et al. 2020).

Finally, during the COVID-19 pandemic in 2020, apart from multimedia and string data types, as for (iii) time series are a common kind of data that arises as an event progresses. Time series forecasting used is literature varies from more traditional data analysis methods, to ML methods like SVM, or DL methods like Multilayer Perceptrons (MLP) or Recurrent Neural Networks (RNN), as, in particular, Long Short-Term Memory (LSTM) and GRU (Gated Recurrent Unit). Other considered AI methods are Polynomial Regression (PR) and Probabilistic neural network (PNN), a supervised feedforward neural network. For careful preparation and distribution of resources, estimating the number of infections is essential, so in the field of forecasting data using time series analysis, an important model is Autoregressive (AR), Integrated(I), Moving Average (MA) or a combination, like ARIMA. However, ANN has been recognized as effective for univariate time series data. To successfully model the spread of the infection and estimate the future number of infections in the population, researchers used RNN and their variant LSTM.

**Fig. 4:**
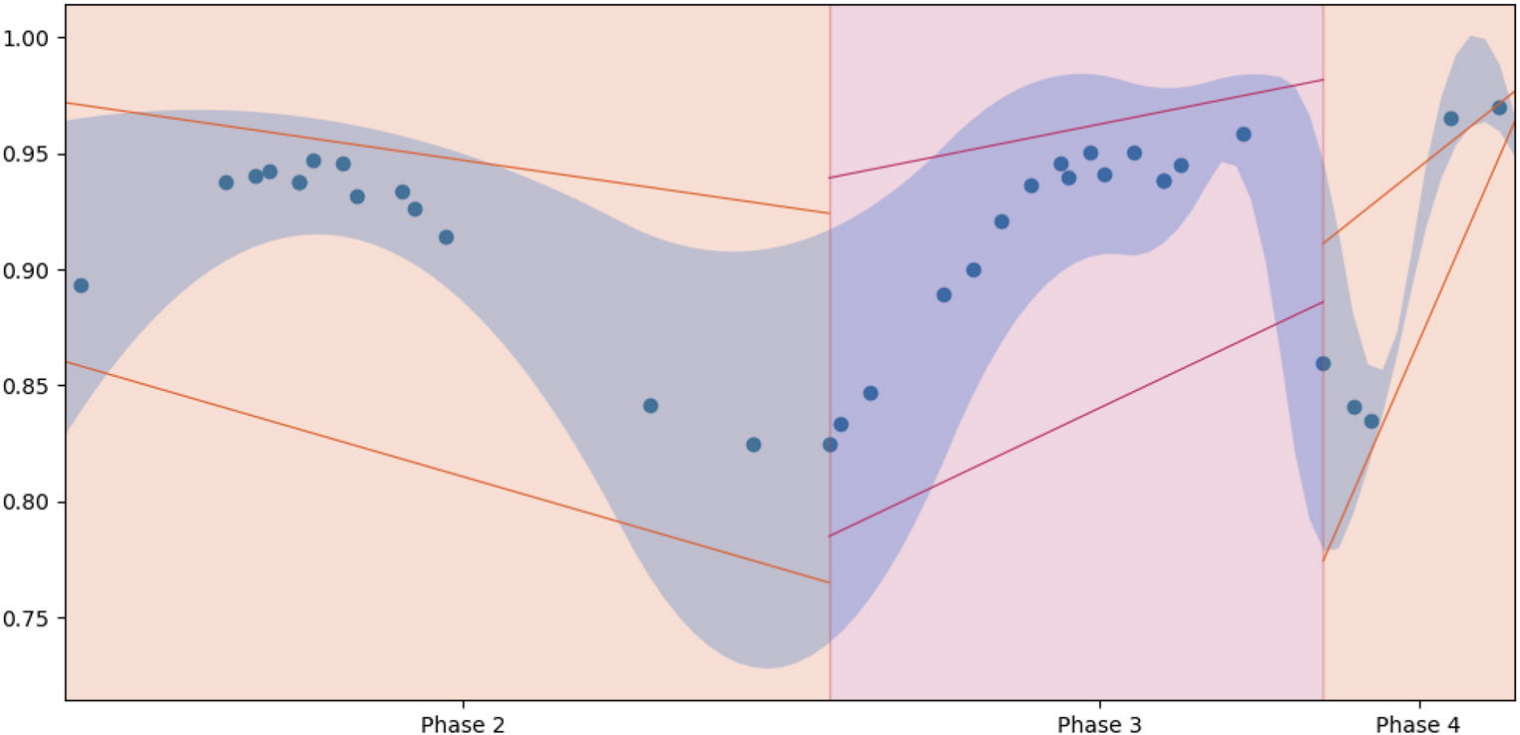
The ROC-AUC trend. Calculated by analysing best and worst classification ROC scores reported into COVID-19 related papers. For each phase, the trend of the best and worst values are calculated and mean trends fitted with a spline, and by showing the papers reported in our tables that reported AUC values

To better observe how proposed research works have improved performance in classification tasks, we plotted the Receiver Operating Characteristics Area Under the Curve (ROC-AUC), better known as AUC, reported in those works. The AUC offers an overall performance metric, that indicates the chance that the model will rate correctly true positive and false negative occurrences. The plotting in Fig. 4, reports on the *y* axis the AUC measure, in average and min-max variation, found in analyzed literature, while the *x* axis represents the positioning of such manuscripts with respect to the temporal line, taking into account the proposed division in phases of the pandemic. This two variable analysis made us investigate the performance of classification tasks as the pandemic progressed.

The *AUC* trend shown in Fig. 4 depicts how qualitative behaviour of published models improved during the pandemic, in terms of classification performances with respect ot AUC. In Phase 2, papers mainly analyse known models on the first bench of data. The AUC decreases due to the exploration of new techniques that require a tuning step. In Phase 3, based on explorations conducted during Phase 2, models become more robust, and min a max AUC values come closer, and optimal AUC values emerge. In Phase 4, challenging models are implemented, and accuracy can easily increase since a wide spectrum of data is coming, while fine-tuning procedures have become more feasible.

### Trends and Directions

In our view, some advice emerges from the literature review that researchers working in DL/ML contexts might apply in developing strategies to fight the COVID-19 pandemic.

As for multimedia classification, the use of Grad-CAM techniques should be recommended and used as best practice in the development of DL techniques, especially for medical diagnostic problems. In fact, as more sophisticated neural network architectures arises, their usefulness remains constrained by their undebuggability and their failure to justify their decisions for humans. Explainable AI (XAI) will be one of the main tools in dealing with what remains a big obstacle for future AI, the “black-box approach”, as to develop explicative models to address real-world issues, in order to improve human understanding of artificial intelligence (Goebel et al. 2018).

As for drug repurposing and clinical trials, AI should be an extension to human workflows, done in multidisciplinary teams so to guarantee that ML/DL strategies are tailored to specific scenarios. Furthermore, the complexities of designing AI solutions for molecular generation must be developed and strengthened, as well as best practice for data exchange (Levin et al. 2020). As for drug repurposing and clinical trials, while there are overlaps between computationally predicted drug repurposing and clinical researches, despite the promising potential of AI applications on this topic, we found no concrete evidence that clinical trials were performed based on computational guidance. Up to our understanding, DL predictions in the majority of the studies analyzed were not followed-up by clinical confirmation. Computational methods should produce promising sets of candidates for potential pandemics, which can then be tested in trials or screenings. However, there is still more research to be done in this area.

As regards text analysis applications, most of them used NLP techniques combined with some specific fine-tuned method for COVID-19 related data and then showed the generated insights extracted from such data. In our opinion, research dealing with string patters did not bring any novelty either in drug repurposing or test analysis.

For time series analysis, in general, considering research papers involving COVID-19 predictions, there is no common agreement in the kind of model to use. The predicted number of deaths due to COVID-19 is not constant, and all the forecasting methods have revealed a large variety of differences in forecasts. Unfortunately, the lack of data related to COVID-19, especially at the beginning of the pandemic, and the changes in policies make it more important for research that learns from small datasets or improves the accuracy of mathematical models (e.g. the SIR model), that predict reduced-size data. In general it does not exist a method that performs better than others, and it depends, on the prepossessing, and fine tuning. A best practice that we advise is the use of data fusion techniques, to mix time series coming from multiple data sets, as in Kim et al. (2020) and Ou et al. (2020).

### Cross-cutting Aspects

Let us look at the fundamental and cross-cutting causes of potential AI failure in terms of its application in relation to a pandemic.

Firstly, although several unique implementations have exceeded the analogous human capabilities, there is no single AI method that can replicate or improve the human mind. In other words, we currently have only weak AI, not yet strong AI. By focusing on a pandemic, these limitations fragment the assistance that technology can offer. The more specific the applications are, the less they can achieve a complex and challenging task. Perhaps, future research on more complex NNs will solve such problems, like deep quantum NNs (Beer et al. 2020).

Secondly, AI does not function without knowledge of the domain. For example, concerning the identification of diagnostic images, it consists of an exercise in obtaining greater accuracy without a radiologist’s assistance. In the same way, the different application fields need to be known. An interesting benchmark relates to the case of Ebola in Sierra Leone. The research team assumed that a disease such as Ebola was malaria-like, spread when people travelled, and the smartphone tracked people moves. Unfortunately, in these countries, it is usual for many people to share the same phone (Erikson 2018), and so a potentially interesting strategy led to inconsistent results.

Thirdly, let us consider the data. AI cannot operate unless powered by a sufficient flow of data, both quantitatively and qualitatively. Luckily, there are not millions of pandemics, but only a few. Regarding past pandemics, data are not collected in a structured, systematic, and time-dependent manner, as in the case of COVID-19. It reveals a weak point of AI methodologies: disease studies utilise rare occurrences to predict rare occurrences. In contrast, ML uses a large amount of data to predict common occurrences (Geoghegan and Holmes 2017; Fountain-Jones et al. 2019). It means that the best results are thus obtained in the presence of repeated events occurring with normal parameters. Besides, variety in the methods of gathering information contaminates the consistency of the information. Activities that involve attempts to look for flawed patterns, for example, concerning swabs collected from different groups of patients, become troublesome for researchers who study trends. Therefore, how appropriate AI’s application is in relation to such problems is a question that arises spontaneously.

### Ethical Aspects

The new society’s nature, intertwined with AI after the pandemic, is fraught with problems, especially about ethical issues, such as trust, accountability, and privacy. When an algorithm fails to diagnose a health issue, what needs to be taken into account? Lack of trust in AI is a widely-known concern, which has emerged anew during the COVID-19 pandemic. Despite their undeniable potential advantages, frameworks for medical diagnosis based on deep NNs may pose problems in comprehension and transparency concerning prediction. DL’s advent is one of the significant reasons why interpretability has become a much-discussed topic in the last decade. Issues include the assignment of responsibility for making a bad diagnosis and the limitations in knowing how the NN has generated such results, namely the *black-box problem*. Explainable AI (XAI) can help to solve some of these issues, but careful research is required to find suitable solutions (Heinrichs and Eickhoff 2020).

Trust in AI also involves trust in researchers and the papers they write. Unfortunately, although there has been a wide range of papers published during the first few months of the COVID-19 pandemic, these have not always been accompanied by a similar quality level. Such works were sometimes clogging up research journals and making it more difficult to find valuable insights.

Moreover, another significant ethical problem is privacy, for example, in relation to contact-tracking apps that need to balance the efficacy of public health protection with the safeguarding of civil rights (Sweeney 2020; Ahmed et al. 2020), or wearable devices, that could pave the way for some form of mass surveillance during a pandemic. On the bright side, if the public is prepared to surrender some privacy, AI systems can be extremely helpful. The challenge will be to find the correct balance between privacy and services. When these services are health-related, people will generally prefer health over privacy (Harari 2020). However, this could mean that an authoritarian government can take advantage of any pandemic and maintain temporary restrictions for a longer time than is strictly necessary. Furthermore, the issues regarding trust and privacy need to be taken into account by policymakers since ethical principles for AI require a definitive regulation (Mittelstadt 2019).

Governments play a critical role in enabling AI to reveal its capacity to assist in tackling a pandemic. It is a lesson we have learned already, from the SARS epidemic in 2002. At that time, although the WHO could track outbreaks of diseases with its GOARN (Global Outbreak Alert and Response Network) system, governments had already amassed considerable powers to conceal diseases and disseminate misinformation throughout their territories, making it a matter of fortune. According to WHO, luck played a central role in the not spreading of the outbreak in Hong Kong, rendering global containment possible (Honigsbaum 2020).

### Conclusion

In conclusion, this work presents a data science perspective on how AI is fighting the COVID-19 pandemic. We have proposed a temporal step approach, describing recent research studies, analyzing how AI observes and acts on the society and health care system and reporting the different data types. Today’s pandemic might bring to light solutions that can help our society, even if AI research has so far proven useful only in a small set of tasks, such as image diagnostics. However, there is still time for research improvements to enhance the current pandemic’s overall AI support. We hope that advances in the future contribution of AI to healthcare systems and society can start from the discussions presented in this paper, taking into account the experiences and limitations of the different solutions provided by the research papers presented in this work.

## Footnotes

1 http://www.scopus.com
